# Characterizing elemental, equivalent black, and refractory black carbon aerosol particles: a review of techniques, their limitations and uncertainties

**DOI:** 10.1007/s00216-013-7402-3

**Published:** 2013-12-03

**Authors:** Daniel A. Lack, Hans Moosmüller, Gavin R. McMeeking, Rajan K. Chakrabarty, Darrel Baumgardner

**Affiliations:** 1 NOAA Earth System Research Laboratory, 325 Broadway, Boulder, CO 80305-3337 USA; 2 Desert Research Institute, Reno, NV USA; 3 Droplet Measurement Technologies, 2545 Central Avenue, Boulder, CO 80301 USA; 4 Cooperative Institute for Research in Environmental Sciences, University of Colorado, Boulder, CO 80309 USA

**Keywords:** Aerosols, Particulates, Chemical sensors, Optical sensors, Thermal methods

## Abstract

Elemental-, equivalent black- and refractory black-carbon are terms that have been defined in order to dissect the more general term, black carbon, into its component parts related to its specific chemical and optical properties and its impact on climate and health. Recent publications have attempted to clarify the meaning of these terms with respect to their environmental impact, particularly on climate. Here, we focus on the measurement aspects, reviewing the most commonly implemented techniques for the direct and indirect derivation of black carbon properties, their strengths, limitations, and uncertainties, and provide a non-exhaustive bibliography where the reader can find more detailed information. This review paper is designed as a guide for those wishing to learn about the current state of black carbon measurement instrumentation, how calibration is carried out, when one instrument may have the advantage over another, and where new techniques are needed to fill important knowledge gaps.

## Introduction

The title of this manuscript specifically avoids the use of the simplified term “black carbon (BC)” in order to emphasize that BC has been used for years as a catch-all term to describe a variety of types of carbonaceous particles. These types of particles are an important and ubiquitous component of the atmosphere that impact climate because of their direct interaction with solar radiation. Some fraction of them indirectly modify climate through their activation as cloud droplets or ice crystals whose size make them more effective dispersers of radiation than when they are aerosol particles [1, 2]. Their emissions from sources of combustion, man-made or through natural events, can modify the weather inadvertently by increasing the number of small water droplets while decreasing the production of rain or snow, an event that is still largely not understood yet generally accepted as an important factor in changing precipitation patterns [3, 4].

Only very recently, within the last 10 years, have researchers started identifying which types of aerosol particles have the largest impact on health. The general correlation between particle mass and mortality/morbidity has been well documented but more recent studies have established a causal link between carbonaceous particles and cardiovascular, respiratory, and neurological problems, although a great deal more research is needed to understand which properties of the particles are responsible for inciting physical disorders [5].

The need to quantify and document the properties of carbonaceous particles has led to the development and proliferation of a number of different instruments that measure these properties using a variety of techniques. Some of the older of these have been extensively evaluated, whereas the newer ones are still being studied to better understand their limitations and the uncertainties associated with their measurements. There are also numerous technical articles that describe the operating principles of these instruments as well as a number of studies comparing one technique against another.

Given the increasing number of investigators in a broad range of scientific disciplines who are interested in the measurement of carbonaceous particle properties to support their research in climate change, air quality, glaciology, health, and in other topics where these type of particles have significant impact, it seemed timely to write a succinct review of the most commonly used instruments that measure these particle properties. This presentation is meant to be a guide to those who are new to this field of research and wish to learn the basic operating principles of the instruments, their uncertainties and limitations, and to find literature references where more detail may be unearthed.

## Definitions

Carbonaceous particles have many different properties but we will focus here only on the properties of those particles that are produced from combustion of fossil fuels and biomass and their physical, optical, and chemical properties. Because of the confusion over the years concerning terminology, we will start with definitions that have recently been clarified by Petzold et al. [6] with respect to the types of carbonaceous particles.

### Black carbon (BC)

Following Petzold et al [6], we use BC when describing the material identified through the characteristic aggregate morphology of combustion sourced particles. Very little qualitative information on mass is achieved using morphology, and so the general qualitative term BC has been maintained for morphologic methods. This term has historically been used to describe those refractory carbonaceous particles that strongly absorb light at all solar wavelengths as reflected in a recent EPA report to congress [7] stating “In this report, BC is defined as the carbonaceous component of particulate matter that absorbs all wavelengths of solar radiation;” where all wavelengths of solar radiation corresponds to “the solar wavelengths present in the troposphere (eg, 280–2500 nm).” Bond et al. [8] provide a refined definition as *“a distinct type of carbonaceous material that is formed primarily in flames, is directly emitted to the atmosphere, and has a unique combination of physical properties.”* Bond et al. [8] and Petzold et al. [6] describe four fundamental physical properties of BC:

1. Strong visible wavelength-independent light absorption with a mass absorption coefficient (MAC) of at least 5 m^2^ g^–1^ at 550 nm
2. Refractory with vaporization temperature near 4000 K
3. Graphitic SP^2^-bonded carbon with aggregate morphology
4. Insolubility in water and common organic solvents

Petzold et al. [6] recommend that ‘BC’ should be used only as a qualitative and descriptive term when referring to light-absorbing carbonaceous particles and should be avoided when describing measurements with evolved gas methods. Given that the term BC has been so widely used by the modeling and assessment communities, its use will be unavoidable but some mitigation is possible as long as additional description is given as to how it is measured.

### Elemental carbon (EC)

This is the component of carbonaceous particles that is thermally stable in an inert atmosphere up to approximately 4000 K. It can only be oxidized at temperatures >340 °C [9]. EC can be derived from evolved gas analyzer (EGA) measurements, aerosol mass spectroscopy, and Raman spectroscopy measurements [6].

### Equivalent black carbon (eBC)

A number of commercial instruments that measure the absorption coefficient of absorbing particles derive a mass concentration of “BC” using a conversion constant referred to as a mass absorption coefficient (MAC). In order to clarify that what is being measured may not be 100 % BC, Petzold et al. [6] recommend the use of eBC when reporting the carbon mass derived from the absorption coefficient.

### Refractory black carbon (rBC)

The carbon mass derived from laser induced incandescence (LII) is referred to as refractory black carbon since it is derived by measuring the thermal emission of the carbon component of the particle that absorbs the laser energy.

### Light absorbing carbon (LAC)

The carbon component of atmospheric aerosol that strongly absorbs light at visible wavelengths [10, 11], including eBC and brown carbon.

### Organic carbon (OC)

This is the component of carbonaceous particles where the carbon molecules are chemically combined with hydrogen and other elements like oxygen, sulfur, etc. [12]. OC can be derived from several different methods and is also an operational definition for EGA measurements.

### Total carbon (TC)

This is the sum of OC and EC derived from EGA measurements.

### Brown carbon (BrC)

The light-absorbing OC in airborne aerosols of various origins, which tends to appear brown rather than black [10, 13, 14]. Their color is a result of non-uniform absorption over the visible wavelength range.

## Measurement techniques

Throughout the remainder of this review, the focus will be on the measurement of EC, LAC (as a pathway to eBC), eBC and rBC. The general structure of the presentations for each measurement technique is (1) a brief description of the measurement principle, (2) discussion of how the principle is applied in practice, (3) cautionary notes on the limitations and uncertainties associated with the technique and interpretation of the measurements, and (4) a list references for further reading (these are combined in the reference section at the end of the paper).

### Elemental carbon (EC)

#### Thermal and thermal optical analysis

Thermal analysis techniques, commonly referred to as evolved gas analysis (EGA), require the deposit of the sample of interest on a filter or aluminum substrate. This is placed in an oven where it is heated over a period of time, in different stages, where each stage represents a different temperature. The property of EC that is being used as a measure of its concentration is its high oxidation temperature, >470 °C, compared with the lower temperatures that will volatilize OC and inorganics. Depending on the particular technique that is implemented, the heating is done in an oxidizing or an inert atmosphere where the number of stages and the temperature at each stage also depends on the protocol that is selected. The word “protocol” is used here in the context of what different monitoring programs have selected as optimum setting for analysis of filters taken at the stations within their network. In the US, the two most commonly used protocols are the National Institute for Occupational Safety and Health (NIOSH) Method 5040 [15, 16] and the Interagency Monitoring for Protected Visual Environments (IMPROVE) method [17, 18], respectively. Other countries’ monitoring programs have implemented similar protocols.

**Fig. 1:**
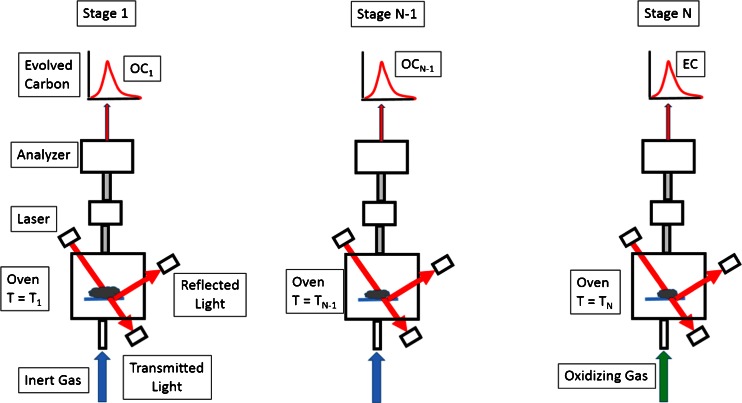
This diagram illustrates that the derivation of organic and elemental carbon using thermal optical analysis requires multiple stages of heating at different set temperatures and with different carrier gases

The implementation of the thermal analysis is shown in Fig. 1. It illustrates the basic steps for achieving a separation between OC and EC. Steps 1 through N-1 are designed to remove the carbon molecules associated with the OC by heating the sample in a non-oxidizing atmosphere so that the OC is volatilized and converted to carbon dioxide (CO_2_) as it passes over a manganese dioxide (MnO_2_) catalyst and is then measured, either with a CO_2_ analyzer or converted to methane and quantified with a flame ionization detector (FID). The last step substitutes an oxidizing gas for the inert gas so that the remaining carbon combusts. Although this measurement can be made in just two steps, N is usually greater than two because OC will volatilize at varying temperatures, depending upon its source and composition; hence, providing additional information about the evolved OC at different temperatures. The laser shown in the Fig. 1, and the associated detectors, are used to make corrections to the measurements attributable to problems inherent in the techniques that are described below.

The advantage of this technique is that the measurement principle and its implementation are straightforward; however, there are a number of limitations associated with its implementation that complicate the interpretation of the results and introduce uncertainties that cannot be completely minimized. An excellent discussion of these limitations and uncertainties can be found in Watson et al. [19].

The separation of EC from OC would be simplified if there were a distinct temperature below which no EC would combust and above which no OC would volatilize. This is not the case since the process of heating the OC can cause some fraction of it to char by pyrolysis. This pyrolyzed carbon will no longer volatilize and, instead, combusts during the final temperature stage, incorrectly being measured as EC. In an attempt to determine how much of the OC pyrolyzes, the sample on the filter is illuminated with a laser and the amount of light transmitted or reflected is measured. The EC on the filter will absorb some of the incident light and as the char is formed it will also absorb light so that the measured transmission or reflectance will be seen to decrease. During the final stage of heating, the pyrolyzed OC and EC will begin to combust, removing the light-absorbing carbon so that the transmission or reflectance is seen to once again increase. When the measured transmitted or reflected light returns to its initial value, it is assumed that all carbon combusted after this point is only EC. The two techniques using transmitted or reflected light are referred to as thermal optical transmittance (TOT) [16, 20] or thermal optical reflectance (TOR) [18, 21], respectively. The analytical limit of detection for these two techniques is about 0.2 μg cm^–2^ [17, 20].

There are various interferences that introduce uncertainties into the determination of the exact temperature at which only EC is being measured. Some EC can be evolved during the inert gas stage as a result of the presence of various metal oxides (eg, Fe_2_O_3_) that will oxidize the EC [22]. In addition, complicating the interpretation of the TOT or TOR is that not all pyrolyzed OC will absorb at the wavelength of the laser [23]. There are additional problems related to the location of the temperature sensor in the oven with respect to the sample since the amount of time the oven is kept at a constant temperature is dependent upon how quickly the sample reaches the set temperature.

The calibration of this method can only be done to determine the response of the analyzer to a known quantity of an OC like sucrose that is put onto a filter, weighed, and then analyzed in the instrument. There is no generally accepted method for calibrating the response of the thermal method to EC since the community has yet to agree on a standard reference material (SRM) [24]. The general features of the thermal analysis measurement technique are listed in Table 1.

**Table 1:** Thermal optical analysis technique summary

Measures	EC
Units	Mass
Collection media	Filter substrate
Collection time	Hours
Uncertainty	±20 %-50 %
Calibration	Currently no generally accepted method to calibrate EC. It can be calibrated to model compounds but there is no generally accepted method for calibration to atmospheric EC.
Biases	Pyrolysis, inorganics
Measures BrC	No

For further reading: (1) experiments that have compared the different thermal methods: [23, 25–30]; (2) interferences from inorganic material: [31–33]; (3) sources of other limitations and uncertainties: [25, 28, 34–36].

#### Raman spectroscopy

Raman spectroscopy (RS) measures the inelastic scattering of light when the vibrational mode of a chemical bond shifts the wavelength of some of the incident light. RS is very selective towards the hexagonal lattice structure of SP2-hydridized carbon, although a range of vibrational modes around a peak energy is observed, RS provides sensitive information on the structural order of atoms within systems that can show crystalline (ordered) or amorphous (disordered) properties and is very selective towards the hexagonal lattice structure of SP^2^-hydridized carbon [37]. When a long-range order of these hexagonal lattices exists, the material is referred to as graphite. The solid carbon produced from combustion of fossil- and bio-fuels results in short range (or disordered) hexagonal lattices [37]. Carbon–carbon (C–C) bonds within an intact hexagonal lattice produce characteristic RS modes at 1585 cm^–1^ (referred to as the ‘G’, or graphitic, mode), whereas C–C bonds at the edges of crystals (incomplete hexagonal lattice) produce RS modes at 1620 cm^–1^ and 1360 cm^–1^ [38–40] (‘D’, or disordered, modes). Because SP^2^-hybridized carbon contains only C–C bonds, these modes are highly selective to the occurrence of just EC.

Subtle differences in the RS modes can provide detailed information on the size and morphology of graphite crystals, and therefore, some contend, can provide source attribution, as morphology can change with combustion conditions [37, 41]. The modes, however, can also change because of other materials within the sample matrix. This is potentially an issue for combustion-sourced EC where common co-emitted species (such as organic matter) contain varying ratios of SP^2^- and SP^3^-hybridized carbon continuum [37, 39, 41]. Commercially available graphitic and organic materials are available to provide reference RS spectra [39, 42], some which show similarities to those of atmospheric EC [42].

**Fig. 2:**
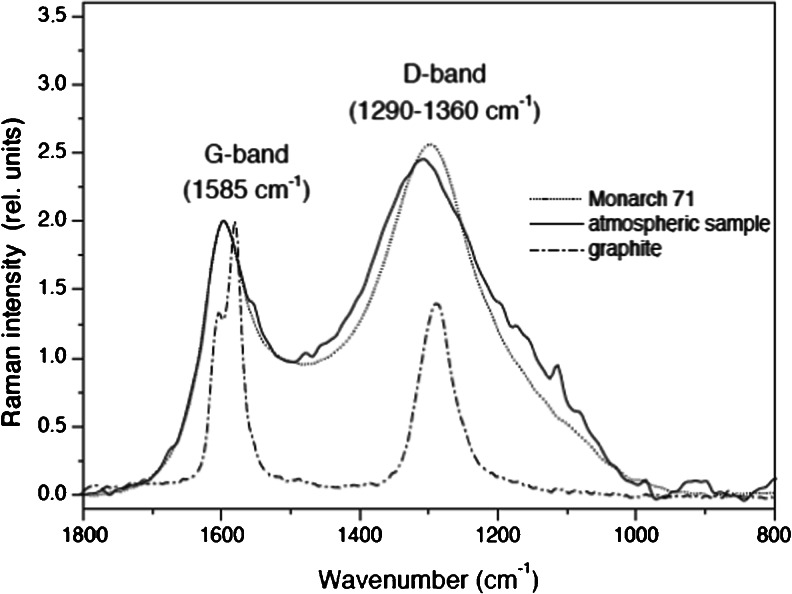
Figure 1 from Mertes et al. [42]. Raman spectra of graphite (dashed dotted line), Monarch 71 (dotted line), and GC contained in atmospheric aerosol particles (solid line)

**Fig. 3:**
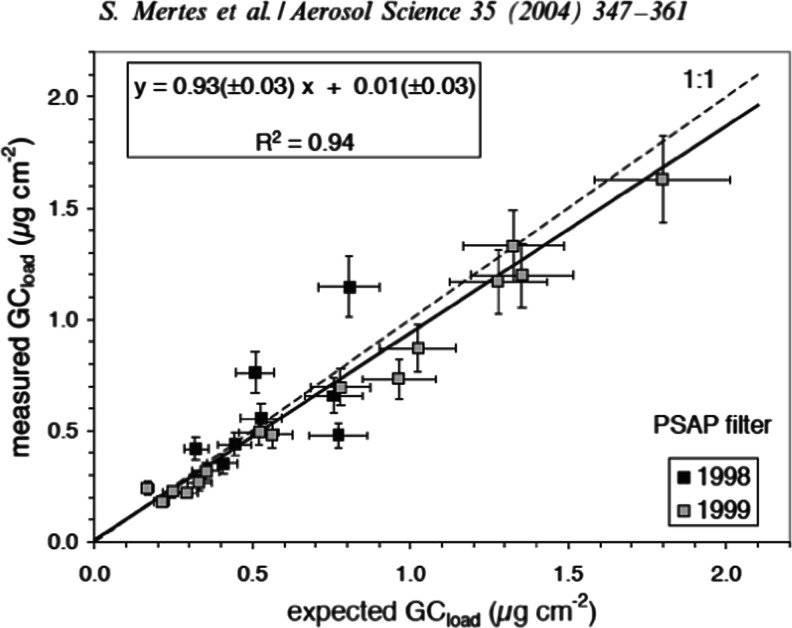
Figure 4 from Mertes et al. [42] comparing GC mass loadings on the PSAP glass fiber filters and simultaneously operated polycarbonate filters from ambient sampling. The expected GC_load_ values (x-axis) are calculated from the GC mass loadings measured on the polycarbonate filters. Regression results (straight line) and the 1:1 line (dashed line) are indicated

The G mode intensity scales with crystal size [39], light absorptivity [43], and mass of EC [42, 44] in the bulk sample and so there is potential to use RS to quantify the absorptive properties and mass concentrations of EC. Rosen et al. [43] presented a semi-quantitative relationship of RS signals and light absorption. They integrated the area of the RS modes around 1600 cm^–1^ and compared this to the light transmission through a filter, which can be corrected to produce a measure of absorption. Keller and Heintzenberg [44] used the ratio of intensities of the modes 1601 cm^–1^ and 888 cm^–1^ to linearly correlate RS response to graphitic carbon mass. Mertes et al. [42] integrated the area of the RS modes around from 1510 cm^–1^ to 1736 cm^–1^ to quantify the mass of atmospheric EC on a filter. To achieve this, they calibrated the RS responses to known masses of a commercially available calibration material that displayed similar RS properties to atmospheric EC (see Figs. 2 and 3). Uncertainties of up to 13 % in RS derived EC mass were reported [42]. If the calibration material does not match the spectral features of atmospheric EC, reported uncertainties will be larger. Ivleva et al. [41] and others have reported that organic components can show RS modes at around 1500 cm^–3^, which may interfere with the wavenumber integration range used for the quantification of EC by Mertes et al. [42]. Organic material can produce high background fluorescence in RS, which can influence curve-fitting routines and limit the quantitative ability of the approach [41]. Ivleva et al. [41] did show a robust negative correlation between the relative content of EC to total carbon and the FWHM of the D band at 1350 cm^–1^ with organics appearing as an outlier to this relationship, potentially providing a semi-quantitative measure of the influence of organic material to the RS analysis of EC mass.

Overall, the RS technique, although providing very specific identification of EC in a sample, has had limited application to the quantification of EC. One major drawback of the technique is that the analysis of RS spectra usually requires curve fitting to multiple convolved spectral features. To achieve robust fitting high signal-to-noise is required; therefore, a sufficient quantity of sample must be collected [37, 39]. To achieve this for atmospheric samples, tens of minutes to multiple hours [42] of sampling are required making RS an impractical technique for real time atmospheric sampling requiring high time resolution. As mentioned, interference by fluorescence of some materials limits the ability to provide accurate curve fitting and so care must be taken that sample collection is not done using a fluorescing filter medium [39]. Sufficiently high laser power can also damage samples, although signal to noise can be improved with higher laser powers. Care should be taken to balance these trade-offs [39]. The general features of the Raman spectroscopy measurement technique are listed in Table 2.

**Table 2:** Raman spectroscopy technique summary

Measures	EC by calibration
Units	Mass
Collection media	Filter substrate
Collection time	Hours
Uncertainty	>13 %
Calibration	Commercially available material can provide calibration for RS spectra and derived EC mass
Biases	Possibly co-emitted organics
Measures BrC	No

#### Insolubility

At room temperature, EC is an inert substance, insoluble in polar and nonpolar solvents including acids, bases, and organic solvents. While this property is often cited as a defining characteristic of EC [6, 46], very little work has been done to develop this characteristic into a measurement method. For the analysis of OC, solvent extraction with polar and non-polar solvents is often used to extract fractions of OC from filter samples [47–49]. Apple et al. [50] have obtained an upper limit estimate of the EC fraction in atmospheric aerosol sampled on glass fiber filters from the carbon remaining insoluble after a two-step extraction process. While we are not aware of any attempt to further develop this estimation into a quantitative measure of EC concentrations, insolubility has potential for separating EC from OC if a suitable protocol with appropriate solvents can be formulated and tested. The general features of the Insolubility measurement technique are listed in Table 3.

**Table 3:** Insolubility technique summary

Measures	EC solvent extraction
Units	Mass
Collection media	Filter substrate
Collection time	Hours
Uncertainty	Unknown
Calibration	Untested
Biases	Unknown
Measures BrC	No

### Equivalent black carbon via measurement of light absorbing carbon (LAC)

Light absorption by particles has been used extensively to derive a mass of eBC requiring the conversion of the light absorption coefficient to mass, via a mass absorption coefficient (MAC). The MAC of atmospheric air masses containing BC can vary with source and can be highly variable. Bond and Bergstrom [11] provide an extensive review of the literature and concluded that the MAC of freshly emitted BC from fossil fuel combustion is 7.5 ± 1.2 m^2^g^–1^ (550 nm), whereas MACs for a variety of BC-containing air masses can range upwards of 15 m^2^g^–1^.

In recent years, it has been recognized that there are multiple contributors to atmospheric particle light absorption that may alter or bias the MAC of atmospheric BC [10, 11, 49]. BC and mineral dust were thought to be the main particle absorbers until recent research into the intrinsic absorptive properties of organic carbon showed that absorption can occur at some specific short, visible wavelengths (called brown carbon, BrC) [10]. Additionally, the enhanced BC absorption by coatings on the BC (internal mixing) has been demonstrated theoretically [51, 52], in the laboratory [53–55], and field settings [56–58]. Given this, it must be recognized that any assumption that an absorption method is measuring exclusively BC must be questioned. When the contribution of these non-BC absorbers is unknown, and a BC MAC is applied, the non-BC absorption is converted to a BC equivalent, thus the source of the term “*equivalent* BC,” described here and by Petzold et al. [6]. This treatment could bias the derived *eBC* mass high compared with the methods outlined in other sections, particularly for commercial instruments that use a single MAC to convert light absorption to *eBC* mass. There is evidence that for measurement of fresh combustion particles that this bias is within the uncertainties of the measurement methods [8, 58]. For instruments that measure light absorption but derive a BC mass by applying a single MAC to all air masses, care must be taken to determine whether the manufacturer applied MAC is appropriate.

These biases may be reduced by determining individual contributions to total absorption. Contamination from dust can usually be determined by a combination of air mass trajectory modeling, particle size measurement or absorption wavelength dependence [53, 59, 60]. Contributions of BrC and internal mixing have been determined using a variety of methods [61–63], however, the multi-wavelength extrapolation methods that have often been applied contain significant uncertainties that should be considered before quantitative attribution is reported [62]. An alternative to measuring these contributions is to minimize them by sample pretreatment such as sample heating to vaporize the semi-volatile materials that lead to increased absorption by internal mixing or BrC [57, 62, 64].

The following sections detail the common filter-based and in-situ particle absorption measurement methods and pre-suppose that (a) the contributions of non-BC absorbers are adequately considered, and (b) an acceptable community standard MAC is applied to derive *eBC*.

#### Filter transmission measurements

The darkening of filters loaded with absorbing atmospheric particles is commonly used to measure particle absorption. The intensity of light measured before (I_0_) and after (I) passing through a filter (of thickness *x*) loaded with particles can produce an absorption coefficient of the particle-filter system (*b*_*pf*_), according to the Beer-Lambert law:

$$ I={I}_o{e}^{-{b}_{pf}x} $$

Instruments that use this filter transmission technique determine the absorption coefficient of the system by knowing the surface area of the collection filter (A), the flow rate of air passing over the filter (V), the sample time interval (Δt), and the light intensities at the beginning and end of Δt [46, 65–71]:

$$ {b}_{pf}=\frac{A}{V}\frac{ ln\left(\frac{I_o}{I}\right)}{\varDelta t} $$

True absorption can only be measured if there is no light scattered off the filter matrix that can be interpreted as absorption. This method also relies on the sample layer being thin to avoid multiple scattering effects of radiation between particles [72]. In practice, these conditions are rarely met, so significant effort to minimize or characterize and correct for these artifacts is necessary to produce accurate *b*_*Abs*_ values [eg, 45, 65, 67]. The following corrections are likely necessary for correction of measured transmission to light absorption:

1. Multiple light scattering within the filter:
  Incident light can scatter from the unloaded filter matrix (membrane or fibers) and increase the sample path, *x* [73]. This will be dependent on filter type and optical configuration of the instrument [46, 71, 74].
2. Filter Loading:
  As the filter becomes loaded with absorbing particles, the incident light is absorbed and less light is passed through the filter, which is the basis for the measurement. However, as loading builds, the sample path (x) is decreased, leading to a bias in the calculated *b*_*Abs*_ [66, 71]. This correction is dependent on the amount of absorbing material loaded onto the filter and dependent on the particle optical properties (eg, single scatter albedo (SSA) and particle size [65, 70, 71], which are highly variable depending on source, transport, processing etc. [71, 75–79].
3. Particle Scattering Correction:
  As the filter is loaded with scattering particles, incident light is scattered in all directions. This leads to higher filter reflectance and more opportunities for absorbing particles to absorb light. This correction will be dependent on total particle scattering (shape, size, composition, mass) [65, 66]. For the particle soot absorbing photometer (PSAP), approximately 2 % of particle scattering is interpreted as absorption [65, 70], whereas the choice of the Aethalometer filter introduces somewhat less of a scattering artifact [73].

**Fig. 4:**
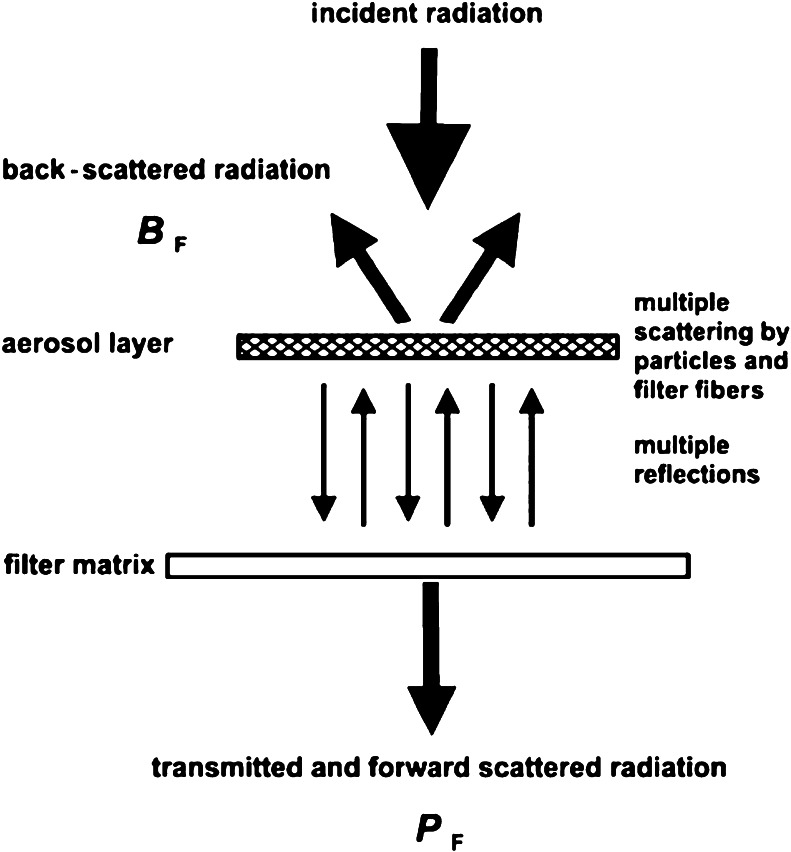
Schematic of the MAAP radiation pathway, highlighting the forward and backscattered radiation, the multiple scattering within the particle layer and filter. Aerosol Science and Technology: Evaluation of multiangle absorption photometry for measuring aerosol light absorption. (39):40–51. Copyright 2005. Mt. Laurel, NJ., Reprinted with permission

In addition to the corrections, these methods require careful calibration of the filter surface area (A), sample flow rate (V) [65, 66, 71], and may have biases due to liquid-like organics spreading across filter fibers [80–82]. This phenomenon introduces one of the limitations of the filter-based methods in that the particles deposited on the filter may change morphology [46], a property that contributes to both scattering and absorption. Additionally, artifacts related to elevated relative humidity and pressure and temperature fluctuations can influence the quality of measurements [83]. See Fig. 4 for a schematic of the radiation pathways for these methods.

There are numerous applications of the filter transmission method. The integrating plate (IP) [68] or integrating sandwich (IS) [84] methods use optical diffusors to measure the scattered radiation to enable scattering corrections. The IP and IS methods also utilize a membrane filter (with a refractive index that is close to that of atmospheric particles) for sample collection and, therefore, minimizes multiple scattering effects inherent to fiber filters. However, because of the sharp filter–sample interface, the filter loading correction can become significant. The IP and IS methods also do not measure continuously, therefore requiring sample collection followed by offline data collection. Detailed discussions on these particular techniques are provided by Clarke et al. [85], Heintzenberg et al. [86], Horvath [87], and Reid et al. [88].

Advances to the IP and IS methods include instruments such as the aethalometer [67, 71], PSAP [65], and the continuous soot monitoring system (COSMOS) [89]. There are also a number of PSAP variants in use; the continuous light absorption photometer (CLAP; US National Oceanic and Atmospheric Administration, Global Monitoring Division), the PSAP-ITM [90], and the spectral optical absorption photometer [SOAP, 91]. These instruments sample continuously (seconds resolution) over multiple wavelengths, and often have multiple filter spots or automated filter changes for continuous field operation. One downside of these methods is the use of fiber filters that introduce varying degrees of scattering corrections larger than those of the membrane filters used by the IP and IS methods. These methods require significant laboratory experiments using a reference absorption method to determine appropriate correction factors [66, 70, 71, 83, 89, 92–94]. Ultimately, these correction factors lead to measurement uncertainties of 20 %–30 % [8]. Of these techniques, the aethelometer and PSAP are the most commonly used commercial multi-wavelength instruments. Collaud Coen et al. [66] and Virkkula and co-workers [70, 94] provide the most detailed discussions on the aethelometer and PSAP, respectively.

It should be noted that the use of these multi-wavelength instruments for interpreting absorption by BC and BrC is not advised, particularly for conditions where BrC does not contribute a significant amount of absorption. Both Collaud Coen et al. [66] and Virkkula et al. [70] point out that there are wavelength-dependent correction factors, whereas Lack and Langridge [62] show the uncertainties of using these multi-wavelength attribution methods.

**Fig. 5:**
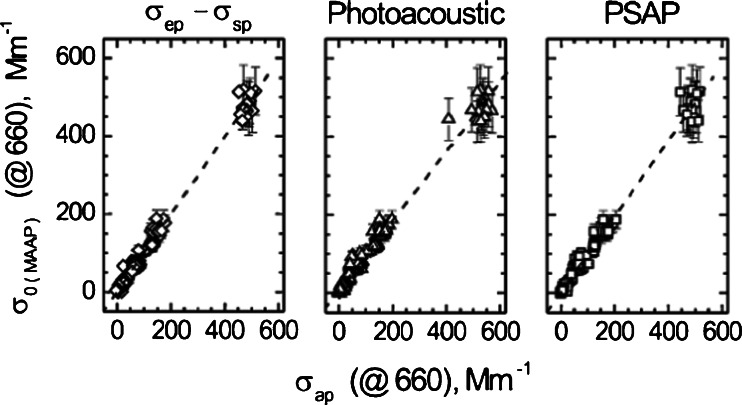
Comparison of MAAP-measured absorption coefficients with those measured by the difference method, PAS and PSAP. 1:1 line shown as dashed line. Aerosol Science and Technology: Evaluation of multiangle absorption photometry for measuring aerosol light absorption. (39):40–51. Copyright 2005. Mt. Laurel, NJ., Reprinted with permission

Recently, Petzold and co-workers [45, 69] introduced the multi-angle absorption photometer (MAAP), which measures, at multiple angles, the back-scattered light that is used in a radiative transfer model to provide the scattering corrections. In contrast to the other methods, the MAAP does not use empirical corrections. Using this method, they reported that filter loading and multiple scattering artifacts were significantly reduced. The MAAP has a reported uncertainty of about 12 % (see Figs. 4 and 5 for the schematic of the MAAP radiation path and comparisons of MAAP absorption with measurements from a PSAP and a photo-acoustic spectrometer (PAS), the latter instrument discussed in the following section.

Although significant work has been done on the measurement corrections for a variety of filter-based absorption instruments, there is still variable quality of *b*_*Abs*_ measured by filter-based methods compared with a *b*_*Abs*_ standard, such as the photo-acoustic method (e.g., [96, 97]), the difference of extinction and scattering method (e.g., 64, 96]), or the MAAP [66, 69, 97]. Controlled laboratory measurements of simplified BC particles or fresh fossil fuel combustion particles usually provide acceptable comparisons, whereas complex particles from a variety of sources can produce deviations from the reference methods [45, 53, 70, 79, 81, 83, 92, 98–103].

We note here that there are other commercial applications of the filter transmission method, particularly in industry applications where the qualitative Filter Smoke number (e.g., [104]), or Bosch number measurements are used to determine exhaust opacity. We note that these measurements, although used under standardized protocols (ISO8178), have not been subjected to the same rigorous artifact corrections as the aethelometer, PSAP, COSMOS, or MAAP.

Advantages of these well-characterized methods include insensitivity to gas phase absorption and simple, inexpensive operation. The disadvantages included the added uncertainty of the measurement because of the required corrections and the removal and potential alteration of the particles from their suspended state. The general features of the filter-based absorption measurement technique are listed in Table 4.

**Table 4:** Summary of measurement features of filter-based absorption

Measures	Absorption and eBC by MAE
Units	Mm^–1^, Mass
Collection media	In situ
Collection time	Seconds
Uncertainty	12 %–30 %
Calibration	Involves corrections requiring extensive laboratory experiments to derive eBC
Biases	Elevated RH, possible elevated levels of OC
Measures BrC?	Multi-wavelength units can provide qualitative to semi-quantitative estimates [62]

#### Photo-acoustic techniques

**Fig. 6:**
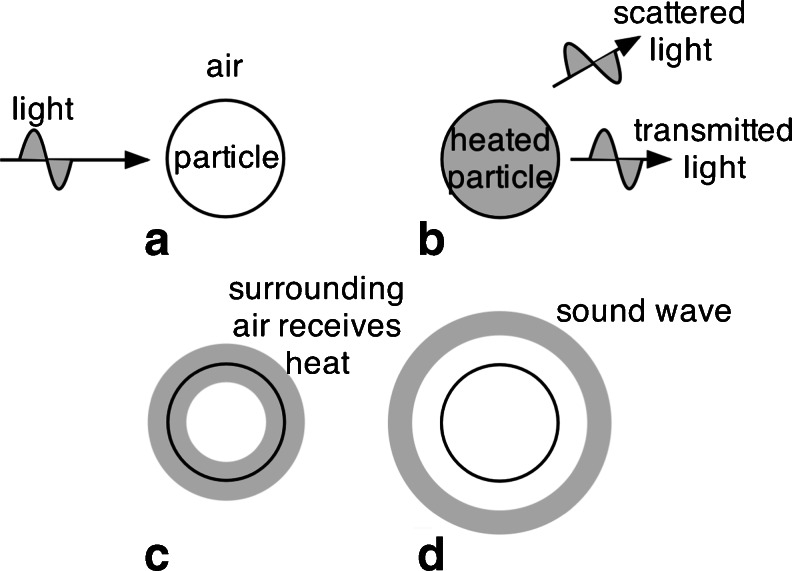
(**A**) Light is incident on a particle. (**B**) Some of the incident light is absorbed by the particle, some is transmitted, and some is scattered. The particle is heated by light absorption. (**C**) Heat transfers from the particle to the surrounding air. (**D**) The surrounding air expands upon receiving heat, resulting in an outgoing acoustic wave if the incident light is power-modulated (from [46])

Thermal measurement techniques, including photo-acoustic and interferometric (see following section) techniques are very direct, in situ measurements of the aerosol absorption coefficient at the wavelengths of the light source(s) employed. They quantify the fraction of absorbed optical energy that is rapidly transferred into the surrounding air as illustrated in Fig. 6 for the photo-acoustic technique [46]. Light, generally in the form of a laser beam, is incident on a particle suspended in air (Fig. 6a); some of the light is transmitted, some scattered, and if the particle has a non-zero imaginary component of the refractive index, some is absorbed and heats the particle (Fig. 6b). For small particles, the heat is rapidly transferred to the surrounding air (Fig. 6c), and if the incident light is power-modulated, an outgoing acoustic wave at the modulation frequency is generated [46]. Generally, an acoustic resonator is employed to enhance the acoustic signal and to exclude and reduce acoustic noise. The resulting acoustic pressure is quantified with a microphone where the resulting signal is proportional to the aerosol absorption coefficient [105].

To calibrate the photo-acoustic technique, the microphone signal needs to be related to the absorption coefficient of the sample. For this purpose, a sample with known absorption coefficient can be employed, for example a gas such as nitrogen dioxide [106], ozone [96], or oxygen [107, 108], which has a well-known absorption spectrum and that can be introduced into the sample volume at a controlled concentration. A related calibration method that does not rely on previously measured absorption coefficients and concentrations introduces an absorbing gas or aerosol with sufficiently high absorption coefficient so that the transmittance and thereby the extinction coefficient can be measured accurately. If scattering can be neglected (generally true for gases), calibration can be achieved by assuming that the absorption coefficient equals the extinction coefficient. For aerosols, scattering cannot be neglected but the scattering coefficient can be measured with the reciprocal nephelometer [109] that is commonly integrated into photo-acoustic instruments and the absorption coefficient can be obtained by subtracting the scattering coefficient from the extinction coefficient. For the measurement of eBC, a correlation between BC mass and measured absorption needs to be established (e.g., [110, 111]). This is best done at a near-infrared wavelength where contributions of brown carbon to absorption are insignificant.

For gases, not all of the absorbed energy may be available as acoustic energy because it may be transferred into different pathways. This must be taken into account if the instrument is calibrated using gaseous absorption. One example of an alternate, energy-absorbing pathway is the photodissociation of the absorbing molecule, thereby reducing the acoustic signal. The photodissociation of nitrogen dioxide (NO_2_), a frequently used calibration gas, is prevalent in the ultraviolet below 398 nm [112]. Another source of energy absorption is collision-induced relaxation that limits conversion of absorbed energy into acoustic energy. This is common for the oxygen molecule [108]. For the case of calibrating with particles, not all of the absorbed energy may be transferred into acoustic energy because (1) part of the energy is used to evaporated semi-volatile particle compounds such as water, thereby reducing the acoustic signal [113–116], (2) because for large particles, the time constant of heat transfer to the surrounding air [117] might be larger than the inverse of the power modulation frequency. In the latter case, the error can be detected and quantified by observing the phase shift of the acoustic signal [46]. Gaseous absorption of the aerosol sample will also contribute to the measured absorption coefficient. To obtain the absorption coefficient of the particles, wavelengths where gaseous absorption of air is minimized should be used. Any remaining gaseous absorption can be subtracted by periodically or continuously measuring the absorption of particle free (ie, filtered) air [118].

Several research groups and companies have built and are using photo-acoustic instruments for the measurement of aerosol light absorption. Early efforts were often hindered by the use of very large and power-consuming lasers such as argon ion lasers (e.g., [119]), whereas more recently small and efficient diode lasers and diode-pumped solid state lasers are commonly used (e.g., [118, 120]). Some early efforts used radial and azimuthal resonators [121–123], whereas currently longitudinal resonators of half- or full-wavelength length have become more common [46]. The influence of acoustic background noise has been reduced, for example, by placing sample inlets at nodes of the acoustic pressure [118], employing acoustic notch filters [118], using subtraction techniques employing two identical resonators with only one containing a laser beam [96], and by reducing noise from the sample pump with a critical orifice [46]. Such reduction of acoustic background noise, together with the use of phase-sensitive detection techniques and powerful lasers and optical multi-pass cells [96], has yielded detection limits for the measurement of aerosol light absorption below 0.1 Mm^–1^ (60 s averaging time) and an instrument accuracy of ~5 % [96]. In addition, the ongoing development of multi-wavelength photo-acoustic instruments [124–128] is important for characterizing the wavelength dependence of aerosols such as brown carbon and mineral dust [46].

Photo-acoustic instruments have been used for characterization of aerosols in ambient air with instruments deployed stationary and on vehicles, including airborne deployment [46]. The early characterization and measurement of eBC and the calibration of filter-based measurements (e.g., [80, 81, 92]) have been complemented with characterization of brown carbon [63, 124, 129–132] and mineral dust [133], and the investigation of the role of particle coatings [53, 63, 132, 134], morphology [135], and humidity induced particle collapse [136] on light absorption. While photo-acoustic instruments have become the “standard” for the accurate measurement of aerosol light absorption [97], deployment is still limited compared with filter-based instrument, and part of their utility has been in improving the calibration of filter-based measurements. The general features of the photo-acoustic measurement technique are listed in Table 5.

**Table 5:** Summary of measurement features of photo-acoustic techniques

Measures	Absorption and eBC by calibration
Units	Mm^–1^, Mass
Collection Media	In situ
Collection time	Seconds
Uncertainty	5 %
Calibration	Calibration aerosols and gases
Biases	Large particles (>2.5 um), elevated RH
Measures BrC	At appropriate wavelengths, with appropriate sample conditioning

#### Interferometric techniques

Light absorption can be measured by photo-thermal methods that alter the density and refractive index (RI) of air molecules or particles that absorb laser radiation [137]. Photo-thermal interferometry (PTI^1^) is the method most commonly used to measure particle absorption [138–141] and is the focus of this review section.

When particles absorb radiation, the energy is returned to the surrounding air by collisional quenching, which heats the air leading to gas expansion, gas density changes, and RI changes [141]. These changes are measured using an interferometer, where two identical laser beams (split from the same source) pass through almost identical geometric paths. The only difference in beam paths is that the ‘probe’ beam travels through the sample volume and is then recombined with the second ‘reference’ beam after which an interference pattern is measured. When the sample is heated with a ‘sample’ laser (modulated at frequencies <100 Hz, [142, 143]), the optical path length of the probe beam changes because of the gas density change, which leads to a phase shift between the probe and reference beams, observable in the interference pattern. This phase shift is proportional to the RI change of the sample air, and the amount of energy absorbed by the sample [141, 142, 144, 145].

Theoretically, PTI can achieve detection limits two orders of magnitude lower than PAS [144]; however, early implementations did not achieve such detection limits [96, 141, 142]. This can be attributed to unwanted changes in the optical path length from sample turbulence, temperature gradients, and mechanical vibrations [141, 142, 146]. Signal to noise can be improved by increasing sample and probe laser power and integrated sample volume [141–143], although particle components, including water at elevated RH, can volatilize and create measurement biases (also common to PAS) when subjected to excessive laser power [142, 145]. Probe beam modulation frequency can also be chosen within a certain range to avoid mechanical vibrations, whereas the probe laser wavelength can be chosen to avoid absorption of specific gas or particle species [141, 142, 147, 148].

The PTI signal is linear with high concentrations of absorbing species [141, 145]; however, sample saturation can occur when the phase change between probe and reference beams becomes too large [142] and when the particles are too large to transfer the absorbed energy to the surrounding air within the probe beam modulation period (an issue common to PTI and PAS) [141, 142]. Measurements of sub-micron particle properties and sample dilution can successfully avoid these issues [141, 142, 145].

PTI calibration for particle absorption has been achieved in a number of ways. Lin and Campillo [141] and Sedlacek [143] measured the PTI phase shift of known concentrations of ethylene or NO_2_, utilizing the known absorption cross section of these gases to determine absorption. Particle mass, calculated from known particle diameters and number concentrations of mono-disperse ammonium sulfate particles, rather than absorption, was correlated to the PTI phase shift by Fluckiger et al. [142].

**Fig. 7:**
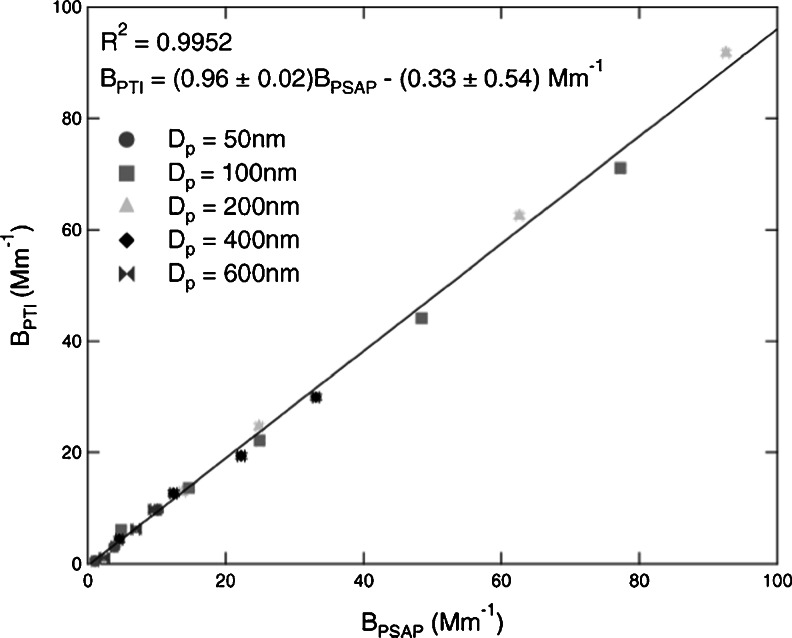
Figure 3 from Sedlacek and Lee [145], plot of nigrosin absorption coefficients measured by the PTI and Aerosol Science and Technology: Photothermal interferometric aerosol 1813 absorption spectrometry. (41):1089–1111. Copyright 2007. Mt. Laurel, NJ., Reprinted with permission

Due to fundamental instabilities in the various interferometer optical designs that make the technique susceptible to mechanical vibrations [141], the PTI technique has seen very limited application for measuring particle light absorption [141, 142]. To overcome this issue, Moosmüller and Arnott [146] introduced a unique interferometer design that virtually eliminated these mechanical instabilities, the folded Jamin interferometer, and this design has proven to be capable of robust visible wavelength light absorption measurements by particles [143, 145, 149]. Sedlacek and Lee [145] showed a 10 % uncertainty in measured absorption and 0.4 Mm^–1^ detection limit (10 s sampling time), in addition to showing accurate comparisons to absorption measured by other techniques (PAS and PSAP). For absorption by particle from laboratory and ambient sources, comparisons of PTI and PSAP to within 4 % were achieved (Fig. 7).

With these recent advances and other advantages over filter-based methods (ie, no particle scattering interferences [143]), and claims of superiority over PAS [142], PTI would appear to be a favorable advance for measuring particle absorption, particularly when multi-wavelength PTI instrumentation seems eminently possible [145]. Despite this, PTI remains a research tool with limited applications for measuring particle light absorption.

Comprehensive treatments of the theory of the PTI technique are presented in a number of studies (e.g., [138, 139, 141, 142]), while the technique also finds common application in metrology [150–153]. Moosmüller et al. [144] provided a review of PTI for particle absorption measurements following the critical advance of the folded Jamin interferometer by Moosmüller and Arnott [146]. Since these two publications, only the work of Sedlacek and coworkers [143, 145, 149] has provided any advance to the science on PTI measurement of particle absorption. The general features of the photo-thermal interferometry measurement technique are listed in Table 6.

**Table 6:** Summary of measurement features of photo-thermal interferometry

Measures	Absorption and eBC by calibration
Units	Mm^–1^, mass
Collection media	In situ
Collection time	Seconds
Uncertainty	10 %
Calibration	Calibration gases of known concentrations
Biases	Large particles (>2.5 um), elevated RH
Measures BrC	With instrument development

#### Remote sensing measurements

At the moment, eBC cannot be derived using remote sensing techniques, although a great deal of effort is being invested in finding the means to do so. If it is possible to remotely measure aerosol light absorption or single scattering albedo (SSA) at multiple wavelengths, it may be possible to distinguish between the dominant aerosol types that absorb in the visible and near-visible wavelengths, more specifically BC, BrC, and mineral dust [154]. The derivation of aerosol light absorption by remote sensing techniques has in itself been challenging with mainly multi-angle techniques showing success. Single-angle techniques such as monostatic lidar [155] or simple sun photometry [156] cannot distinguish the scattering and absorption components of extinction. Advanced high-resolution lidar [157–161] and Raman lidar systems [162–164] can measure aerosol particle extinction and backscatter coefficients; however, the 4π-integrated aerosol scattering coefficient cannot be derived from the aerosol backscatter coefficient without knowledge of the particle-phase function that depends on the size distribution and refractive indices of the ensemble of particles that scatter and absorb the incident light. It has been found, however, that mineral dust aerosol extinction coefficients depend much less on the imaginary part of the refractive index than mineral dust backscatter coefficients; hence, their ratio may provide some indication of mineral dust aerosol light absorption [165]. Multi-wavelength Raman lidar has been used for the retrieval of the SSA for spherical particles [166] and bi-static lidar systems may be able to derive significant parts of the aerosol phase function; hence, there is some potential for deriving light absorption by remote sensing, possibly in combination with sun photometry [167].

The retrieval of aerosol light absorption, using the measurement of diffuse and direct solar radiation [168], is being attempted with multi-angle and multi-wavelength observations of sun and sky radiances with sun and sky scanning radiometers, as implemented by AERONET [169] or multi-filter rotating shadowband radiometers such as those deployed by the ARM program [170, 171]. These retrievals require sophisticated inversion algorithms that are based on models of the vertical profiles of atmospheric particles and optical calculations of their properties for spherical or spheroidal particles. These retrievals yield column-averaged aerosol light absorption, which is commonly given either as SSA or absorption optical depth [172–175]. Spectral SSA data from AERONET have been used to derive concentrations of mineral dust iron and BC during dust and pollution episodes. Some of these retrievals show reasonable agreement with chemical analyses [176]. Measurements of aerosol light absorption with sun and sky radiometer networks are extremely useful for the characterization of aerosol light absorption for specific aerosol types and for determining their atmospheric distribution [154, 177, 178]. However, these observations yield column-integrated measurements and, therefore, “effective” values, potentially including different aerosols and mixing states. Additional comparisons of remote sensing with direct in-situ measurements of aerosol light absorption would be desirable.

The retrieval of aerosol light absorption from satellite measurements is even more challenging [179] because of the spatial and temporal variations of the earth’s surface albedo that often dominates the measured radiances. Proposals have been made to use critical reflectance [180, 181] and sun glint over oceans [182], yet only UV measurements, originally designed for the monitoring of stratospheric ozone, are currently employed operationally, with accuracies that still remain open for debate. Critical reflectance methods and multi-angle measurements are showing some promise, as are novel multi-angle, polarimetric measurements, whose objective are more accurate SSA retrievals.

Sensors that are currently being used to retrieve aerosol optical properties include the total ozone mapping spectrometer (TOMS) and the ozone-monitoring instrument (OMI). TOMS was deployed on board the Nimbus-7 (1979–1992), Meteor-3 (1991–1994), and Earth Probe (1996–2006) satellites. Data products from TOMS include the aerosol index (AI), calculated from the difference in surface reflectivities derived from two UV channels [183]. The AI, although effective for the detection of aerosols above land and ocean surfaces, including the detection of absorbing aerosols (ie, smoke and mineral dust) above ice, snow, and clouds [184], and qualitatively mapping the distribution of aerosol light absorption, does not provide a quantitative measure of aerosol light absorption. The TOMS observations, when used in conjunction with the moderate resolution imaging spectroradiometer (MODIS) [185] measurements in the visible spectrum, have been utilized to retrieve the SSA [186–188]. MODIS observations have also been used to implement the critical reflectance method [180, 181] for retrieving aerosol SSA [189]. The multi-angle imaging spectroradiometer (MISR), operating in the visible-near-IR spectrum, on board the Terra satellite (1999–present), is able to distinguish weakly- from strongly absorbing aerosol types by retrieving the SSA [190–193].

There was the expectation that the state of satellite aerosol light absorption retrieval would be improving after the 2009 launch of the aerosol polarimetry sensor (APS) on board the Glory satellite [194, 195]; however, this satellite experienced a launch failure and never reached orbit. APS data products, based on the multi-angle polarimetric capabilities [194, 196] were to include aerosol SSA in at least three spectral channels for fine and coarse modes with an SSA uncertainty of 0.03. The general features of remote sensing measurement technique are listed in Table 7.

**Table 7:** Remote sensing technique summary

Measures	Light absorption and extinction
Units	Aerosol optical depth
Collection media	None
Collection time	Variable
Uncertainty	Unknown
Calibration	None
Biases	Unknown
Measures BrC	No

### Refractory black carbon

Refractory black carbon is measured using laser induced incandescence (LII). LII occurs when light-absorbing particles are illuminated by intense radiation and heated to temperatures much higher than the surrounding air. The high temperature particles emit grey/blackbody radiation that can be detected and used to derive the mass of the illuminated particle or particles. At sufficient light intensities, particles are heated to their vaporization temperature (or boiling point), which for rBC is approximately 4300 K [197]. At this point, the energy absorbed is approximately balanced by the energy lost via vaporization and radiation. The LII signal decreases as the particle shrinks since less light is absorbed and emitted by the evaporating particle.

Investigations into rBC using LII fall into two sub-groups, those using pulsed lasers and those using continuous lasers. Interference during Raman spectroscopy measurements motivated the earliest LII work on rBC produced in flames [198]. Weeks and Duley [199] showed that LII signals could be related to particle size for carbon black; however, it was the subsequent theoretical and observational studies [200, 201] that showed that LII could be used to directly derive the rBC properties in flames. For example, Melton [200] showed that the magnitude of the incandescence signal could be related to the volume concentration of rBC in the measurement region.

**Fig. 8:**
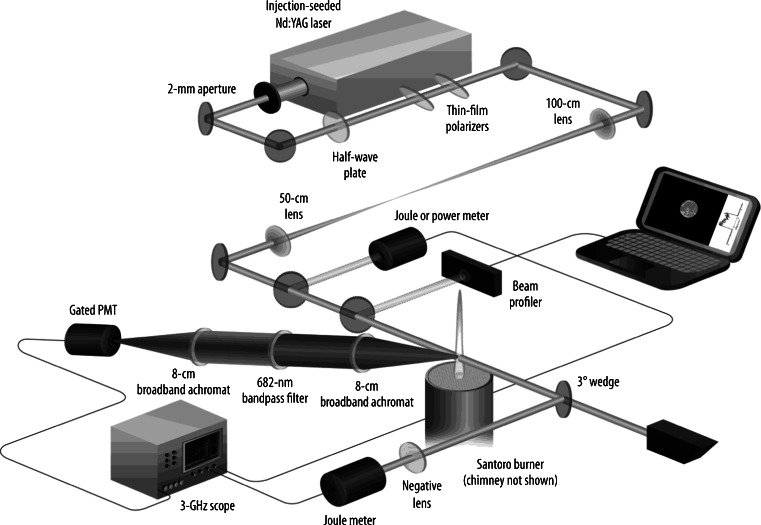
From Michelsen et al. [202]. This schematic is showing the fundamental components of the experimental apparatus. Abbreviations are as follows: B: bandpass filter; CB: camera control box; CL: signal collection lenses; DG, digital delay generators; F: colored glass filter; HV: high voltage power supply; ICCD: intensified charge coupled detector; IL: imaging lend; LWDM: long working distance camera; M: ¼ meter monochromator; PD: photodiode; PMT: photomultiplier tube; SH: laser sheet forming optics; SL: adjustable slit; VCR; video cassette recorder

The LII rBC experiments employ pulsed (~20 ns), high intensity lasers that illuminate a point source or a two-dimensional measurement volume as illustrated in the example shown in Fig. 8 [202]. The ability to visualize the spatial distribution of rBC in the measurement volume was particularly useful for studies of combustion systems (e.g., [203]), particularly those investigating turbulent and/or flickering flames where slower, scattering-based approaches were insufficient [204]. The decay of the LII signal was related to the primary particle size [205] because of the dependence of the particle cooling behavior on surface area. LII was also combined with two-color pyrometry (measuring the light emitted over different wavelength ranges) to determine the temperatures of particles in combustion systems [206]. Pulsed LII has also been combined with light scattering measurements to investigate rBC morphology [207]. Besides rBC measurements in flame combustion processes, pulsed LII techniques were eventually applied to measurements of diesel emissions [208], laboratory-scale gas flares [209], dusts [210], and atmospheric rBC measurements (e.g., [211]).

**Fig. 9:**
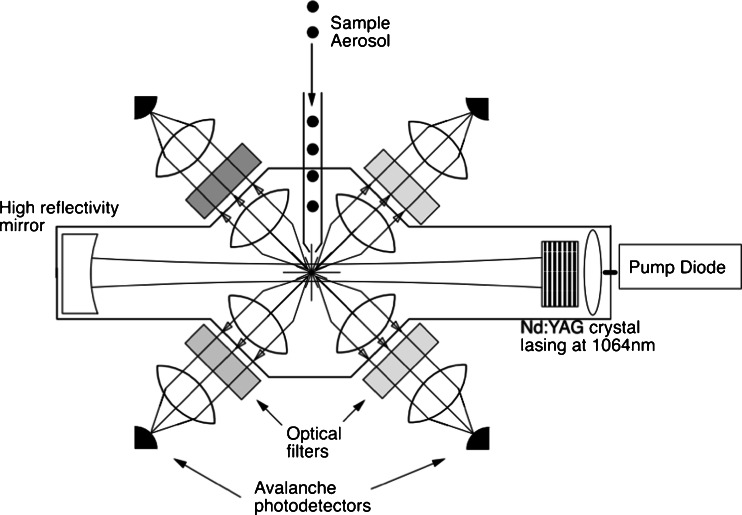
Schematic of the SP2 optical head from Schwarz et al. [95]

Stephens et al. [212] developed an alternative method for measuring LII of single particles for the purpose of identifying particle composition using two-color pyrometry. Unlike previous pulsed LII systems, Stephens et al. [212] illuminated particles by passing them directly through a continuous, intra-cavity, solid-state laser beam. Their design also featured a detector to measure light scattered by individual particles, which was used to estimate their size. Although the main motivation for the instrument was the identification of particle types based on vaporization temperatures, Stephens et al. [212] also highlighted the technique’s ability to measure atmospheric rBC. The design was eventually commercialized as the single particle soot photometer (SP2) and has been used for atmospheric rBC measurements for roughly the last decade. Figure 9 illustrates the configuration of the SP2 for illuminating single aerosol particles and measuring the scattered and emitted components of the light.

The high sensitivity and time resolution afforded by the single particle measurements provided by the SP2 make the instrument suitable for ambient measurements in pristine environments. The earliest applications of the SP2 focused on airborne characterization of rBC in the lower stratosphere/upper troposphere region [95, 213]. The instrument was subsequently widely adopted for not only aircraft-based measurements but also field- and laboratory-based measurements of ambient rBC (e.g., [102, 214–216]). McConnel et al. [217] showed that the SP2 could be applied to measure rBC in liquid samples (ie, melt streams from ice cores), and the approach has since been applied in a number of studies measuring rBC concentrations and properties in ice (e.g., [218, 219]), snow [220, 221], lake water [222], and precipitation [223]. Several investigators have also developed more advanced methods for interpreting the scattering measurements made by the SP2 to infer information regarding coatings and/or other particles mixed with rBC in individual particles [215, 224–226]. These analyses have enabled a number of investigations into atmospheric processing of rBC and the role coatings have on rBC optical properties (e.g., [227]) and hygroscopicity [228, 229].

Uncertainties for pulsed LII measurement methods stem mainly from the need to model the physical behavior of the measured rBC in order to interpret LII signals and determine volume concentrations and size distributions [230]. At high laser intensities, rBC particles vaporize and can undergo restructuring [231], which alters their optical properties and LII signals. Other factors include changes in rBC absorption and extinction properties at different temperatures, expansion of particles as they heat, changes in their refractive index that affect the interpretation of LII signals, and mechanisms through which particles interact with the carrier gas [230]. Measurements of the primary particle size depend on the carrier gas properties and structure of the rBC aggregates. If the aggregate collapses, some primary particles are shielded from the surrounding air, which affects the aggregate cooling rate and subsequent LII signal decay, leading to a larger inferred primary particle size. Chan et al. [211] used this behavior as a diagnostic for rBC aggregate collapse in atmospheric measurements. The majority of pulsed LII studies have focused on combustion environments where coatings on rBC particles are relatively minor. For atmospheric applications aged rBC particles can acquire substantial coatings (e.g., [56, 95]). At high laser intensities, these coatings will evaporate and should not affect measurement of the volume fraction [211]. If coatings or mixed particles survive the initial particle heating, they might affect the determination of the primary particle size from the observed LII signal decay, as they will affect the cooling rate of the mixed particle.

For continuous LII measurements, several studies have investigated the response of the SP2 to different rBC calibration standards, ambient rBC, rBC mixed with other material, and rBC with different morphologies [95, 102, 149, 232–234]. These works have shown that the radiation emitted by rBC particles at their vaporization temperatures is linearly proportional to rBC mass and independent of mixing state and morphology over a wide range of conditions. The lower particle size threshold for the SP2 is limited by the requirement that particles must be heated to their vaporization temperature, and has been found to be 0.7 fg (90 nm volume equivalent diameter) for reasonable laser intensities [235]. The upper size limit of the SP2 is largely governed by the amplification settings on the SP2 thermal emission detectors and the sampling efficiency of large particles to the SP2 detection region. Schwarz et al. [220] have shown that the SP2, when optimized for large particle detection, can adequately measure rBC up to volume equivalent diameters of 2 μm. In most ambient environments, the amount of rBC mass falling outside the SP2 detection range is assumed to be small (10 %–20 %); however, care should be taken when interpreting measurements of fresh emissions where the rBC size distribution may shift to smaller sizes or when examining number distributions where smaller particles make a more significant contribution to total number concentrations compared with total mass. At high particle concentrations, multiple particles can be present in the sensing volume, so care should be taken to avoid interpreting coincident LII signals as originating from a single, larger particle [236].

The choice of calibration material also affects uncertainties in SP2 LII measurements. Different effective densities and emissivities have been found for atmospheric rBC calibration materials, which translate to different LII signals for fixed amounts of rBC mass. Moteki and Kondo [197] showed that combining the SP2 with an independent particle mass measurement downstream of a heated inlet system allowed direct comparison of the LII signal and rBC mass. This work and follow-up studies [234] have shown that rBC in urban environments has an LII response closest to fullerene soot and that the SP2 response to different calibration materials can be related to ambient rBC response using empirical corrections. Similar measurements are needed in non-urban environments to verify that relationships hold for all forms of atmospheric rBC.

Additional uncertainties arise when interpreting continuous LII measurements of rBC present in liquid samples; however, these lie outside the scope of this review. Schwarz et al. [220] is a good starting point for readers interested in more details on uncertainties associated with these methods. Although the identification of coatings associated with rBC also has significant uncertainties, these are not strictly related to the measurement of rBC mass, so we do not include them here. Readers interested in more details are encouraged to consult the original references [215, 224–226].

There are several differences between pulsed and continuous LII methods that we summarize briefly here. Pulsed LII measurements represent the average properties of an ensemble of rBC-containing particles in the sample volume. Particles are illuminated by intense laser light on the order of nanoseconds. The measured parameters are the rBC volume fraction and primary particle size. Two-dimensional LII systems also provide qualitative information on the distribution of rBC within the measurement volume. The technique has mainly been applied to combustion environments, though it is now seeing increasing use in atmospheric applications. The continuous LII method illuminates individual particles on the order of microseconds. The particle mass is determined from the peak LII signal measured when the particle reaches its vaporization temperature. Simultaneous light scattering measurements are used to infer particle-coating properties, whereas measurements of the LII signal over different wavelength ranges are used to infer particle composition. The technique has been used primarily for atmospheric measurements of rBC but is being extended to measure rBC in liquids. Both pulsed and continuous LII methods have uncertainties related to the absorption and emission properties of rBC, particularly when it is heated to high temperatures. It is difficult, however, to extrapolate uncertainties from the two different methods because of differences in how the LII signals are measured and interpreted. That said, we are unaware of any studies that have compared measurements from the two approaches, and much could be learned about both techniques by such a comparison.

A recently developed technique related to LII takes advantage of the selective vaporization that occurs when measuring rBC-containing particle composition using a mass spectrometer. The aerodyne soot particle aerosol mass spectrometer (SP-AMS) is a development of the traditional AMS [237] that replaces the particle vaporization source, a heated metal surface, with the same continuous laser system used in the SP2 [238]. The SP-AMS is not a traditional LII measurement in that the light emitted by the process is not measured. Instead, ions generated from the vapors produced by the LII process are measured to provide detailed chemical information related to both the rBC and any associated coatings. The technique has already been used to investigate atmospheric rBC [56, 238]. A detailed discussion of the uncertainties associated with this technique are beyond the scope of this review, but interested readers should consult Onasch et al. [238] for a detailed discussion of the technique. The general features of laser-induced incandescence measurement techniques are listed in Table 8.

**Table 8:** Laser-induced incandescence technique summary

Measures	rBC
Units	Mass concentration
Collection media	None – in situ
Collection time	Milliseconds to seconds
Uncertainty	5 %–10 %
Calibration	Commercially available light absorbing particles. Fullerene for the SP2
Biases	In the SP2, underestimation of rBC mass if significant mass is in particles smaller or larger than lower or upper size threshold.
Measures BrC	No

### Morphology of atmospheric EC

**Fig. 10:**
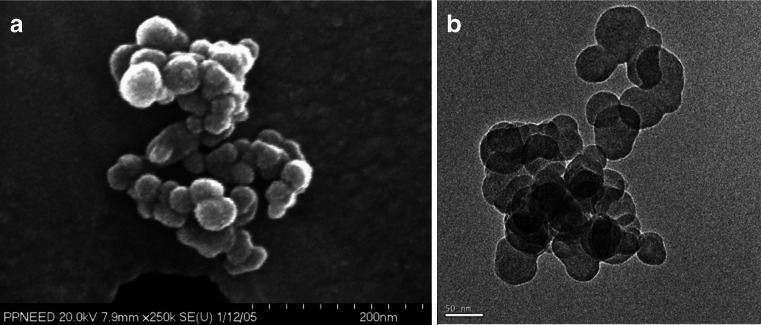
Scanning (**A**) and transmission (**B**) electron microscope images of typical elemental carbon aggregates emitted from high-temperature combustion systems such as engines and biomass burning

Freshly combusted BC particles are emitted as sub-micron sized aggregates of coagulated spherical primary particles (monomers; see Fig. 10) from high-temperature combustion systems [46, 239, 240]. These monomers can lead to varied morphology that BC aggregates can assume in the atmosphere and can significantly affect their physical, chemical, and optical properties. Therefore, experimental techniques to characterize their morphology are of prime interest to researchers.

Transmission electron microscopy (TEM) has revealed the structure of these monomers as containing onion-like layers of graphitic platelets [241, 242], which are not parallel as in pure, single-crystal graphite, but disordered and wrinkled [11]. In the past four decades, laboratory investigations and computer simulation studies have shown that formation of BC in combustion systems proceeds via a three-dimensional diffusion-limited cluster aggregation (DLCA) growth mechanism [243–245]), giving rise to their non-Euclidean particle geometry. Since the application of fractal mathematics to the area of condensed matter physics by Forrest and Witten [246], the morphology of EC aggregates has been described using a quantifiable mathematical parameter, the fractal dimension, *D*_*f*_. Estimation of this parameter leverages from the fact that within a certain length scale, EC aggregates are scale-invariant, that is, their irregularity is similar between the limits of monomer and aggregate size. Mathematically, the number of monomers per aggregate, *N*, scales with the radius of gyration, *R*_*g*_ as [247]:

1 $$ N={k}_0{\left({R}_g/{d}_p\right)}^{D_f} $$

where *k*_*0*_ is the fractal pre-factor and *d*_*p*_ is the average monomer diameter. The aggregate’s *D*_*f*_ is considered to be the key property in influencing its physical, chemical, and optical properties.

Past simulation and theoretical investigations have shown that the process of DLCA always yields an asymptotically converging *D*_*f*_ value of 1.8 [247–249]. However, one often finds the *D*_*f*_ of atmospheric BC aggregates significantly deviating from this value. Freshly emitted BC aggregates often undergo atmospheric processing, resulting in morphologic restructuring [55, 136]. Consequently, these aggregates assume sphere-like, collapsed morphologies with a much higher *D*_*f*_ (close to three).

Practical methods most commonly used for determination of aggregate morphologic properties are in situ light scattering measurements, characterization, and combination of different physical diameters, and quantitative analysis of digitized ex situ transmission/scanning electron microscopy (TEM/SEM) two-dimensional images [135, 239, 240, 247, 250]. In situ measurement of *D*_*f*_ and size of aggregates from angular light scattering measurements involve analysis of scattered light intensity from an ensemble of aggregates in *q* (or inverse length) space [247]. The slope of the linear regions in the intensity versus *q* plots is analyzed to calculate the average *D*_*f*_ of aggregates. The main limitation of this in situ technique is that when using this method it becomes too complicated for extracting the accurate structural properties for polydisperse aggregate size distributions. Furthermore, the instrumentation required for this measurement methodology is often not portable to active combustion sites, making it difficult to study real-world aerosol particles.

Ex situ techniques involving image analysis of aggregates have found wide use in determining *D*_*f*_ of aggregates collected from both laboratory and field studies [135, 239, 251–253]. The Ensemble method (EM) is one of the more popular aggregate morphology characterization techniques used by researchers [239, 253–255]. This technique involves determining the values of *N*, *R*_*g*_, *d*_*p*_ from aggregate images, and then using Eq. 1 to determine the *D*_*f*_ of aggregates. The main drawback associated with this technique is the difficulty of obtaining accurate three-dimensional information about aggregate morphologic variables such as *N* and *R*_*g*_ required for calculating the three-dimensional *D*_*f*_ of aggregates. Although these variables are not directly deducible from a two-dimensional image of an aggregate, empirical relationships between measurable two-dimensional and three-dimensional properties of aggregates have been derived from simulation and experimental studies to remedy this situation and assist researchers in their analyses [256–264].

Over the past three decades, researchers have also employed more simplistic image analysis routines such as the nested squares method (NSM) and the perimeter method (PM), which directly determine the *D*_*f*_ of aggregates from their two-dimensional images. These techniques do not use the relationship between different aggregate properties of Eq. 1 to determine aggregate *D*_*f*_. Determining *D*_*f*_ with the NSM technique involves drawing boundaries (e.g., squares or circles) of increasing size upon a two-dimensional, pixilated image of a fractal aggregate centered on the aggregate center of mass [252, 255]. For every boundary, the number of pixels occupied by the particle is counted. The *D*_*f*_ is calculated as the linear regression slope of the linear portion of the log–log curve generated by plotting boundary size against pixel count. The PM calculates *D*_*f*_ by drawing grids of differing box sizes upon a two-dimensional image of a fractal aggregate [255, 265]. Given a grid with a certain box size, the number of grid boxes through which the perimeter passes is counted. A grid with a different box size is then drawn, and the number of grid boxes through which the perimeter passes is counted once again. This process is repeated, and the logarithm of the box size is plotted against the logarithm of the box count, providing the *D*_*f*_ as the slope.

It is noteworthy to mention that researchers using NSM and PM oftentimes assume that the fractality of the aggregates is conserved between two- and three-dimensions within the aggregate length scale. In other words, the assumption is that the calculated two-dimensional *D*_*f*_ equals the three-dimensional *D*_*f*_. Regarding this assumption, a number of studies [255, 261, 264, 266] have cautioned that factors like orientation of aggregates in the image, location of their center of mass, and the distribution of the primary particles around the center of mass could cause systematic differences between the three-dimensional *D*_*f*_ and the two-dimensional *D*_*f*_ determined from a two-dimensional projection of the three-dimensional structure. Two studies [261, 264] have shown, using computer simulations, that compared to a suspended aggregate, the projected area and length can be overestimated in the two-dimensional projection depending on the resting position of the aggregate on the microscopy filter substrate. More recently, Chakrabarty and coworkers [255] individually tested the accuracies of EM, NSM, and PM in predicting three-dimensional *D*_*f*_ of two-dimensional aggregate images by applying them to a statistically significant (~2500) number of projected images of all stable orientations of computer-generated three-dimensional fractal aggregates with *D*_*f*_ ranging between 1.0 and 3.0. Their results showed that of the three methods, the only method that can be used to reliably determine *D*_*f*_ from two-dimensional images is the EM. Both the NSM and the PM yielded many overlapping values of two-dimensional *D*_*f*_ for differing values of three-dimensional *D*_*f*_ resulting in a non-one-to-one relationship and large margins of error. The general features of morphologic measurement techniques are listed in Table 9.

**Table 9:** Morphology technique summary

Measures	BC structure, fractal dimension
Units	Fractal dimension
Collection media	Nuclepore filter substrate or transmission electron microscopy grids
Collection time	Minutes to hours
Uncertainty	Large uncertainties for nested square and perimeter methods. Low uncertainty for ensemble method
Calibration	None
Biases	Organic carbon coating on BC monomers
Measures BrC	No

## Summary

Recent attention paid to the climate and health effects of atmospheric black carbon (BC) [8, 267] have led to detailed discussions on the general and technical definitions of BC and the terminology of BC derived from various measurement methods. Decades of research and a fundamental complexity in the physical, chemical, and optical properties of BC have led to a variety of ill-defined or confusing terminology and a variety of measurement methods. Bond et al. [8] and Petzold et al. [6] provide general definitions and four fundamental physical property definitions of BC. Petzold et al. [6] further define the terminology of the products of various measurement methods that utilize the physical properties of BC listed above.

In this paper, we have reviewed the measurement methods that correspond to the terminology descriptions of Petzold et al. [6]. Basic measurement principles, advantages, disadvantages, uncertainties, and references for further reading are described. Measurements of the mass of elemental carbon (EC) using the combustion properties of the material, the SP^2^ bonded carbon, insolubility, and the fundamental morphologic properties are discussed. Measurements of the mass of equivalent BC (*eBC*) using the fundamental property of light absorption are provided (filter-based absorption), (photo-acoustic absorption), and (photo-thermal interferometry). A review of the measurement of aerosol light absorption using remote sensing, which requires further scientific advances to derive eBC is provided. Measurements of the mass of refractory BC (*rBC*) using the fundamental vaporization properties are described (laser induced incandescence).

The information that has been put forth in this review was compiled with the goal of helping students, engineers, and researchers in many fields to become better oriented to the many facets of black carbon and to expand their knowledge base of the many techniques that are currently employed to measure these facets.

## Footnotes

1 Here we use PTI for the methods originally described as phase fluctuation optical heterodyne/homodyne spectroscopy (PFLOHS)
